# Effect of different supplements on eggshell quality, some characteristics of gastrointestinal tract and performance of laying hens 

**Published:** 2014

**Authors:** Mosayeb Shalaei, Seyed Mohammad Hosseini, Emel Zergani

**Affiliations:** *Department of Animal Science,Faculty of Agricultural Sciences, University of Birjand, Birjand, Iran.*

**Keywords:** Egg shell quality, Intestinal morphology, Laying hens, Performance, pH value

## Abstract

This study was performed to investigate the effects of antibiotic, organic acid, probiotic and prebiotic supplementation on performance, egg shell quality, pH value of gastrointestinal (GI) tract and small intestinal morphology of laying hens. The experiment was a completely randomized design with 160 laying hens strain (W-36) from 32 to 42 weeks of age, with five treatments, four replicates and eight hens in each replicate. The experimental treatments consisted of: 1-basal diet, 2-basal diet + 150 g per ton antibiotic (oxytetracycline), 3-basal diet + 3 kg per ton mixture of organic acids supplementation, 4- basal diet + 50 g per ton probiotic (protoxin) and 5-basal diet + 2 kg per ton prebiotic (mannan oligosaccharide). During the experimental period, performance characteristics were evaluated. At the end of experiment two birds per replicate was sacrificed for small intestinal morphology. The results showed that organic acid and mannan oligosaccharide significantly increased average egg weight. Also feed conversion ratio significantly improved by mannan oligosaccharide. Eggshell quality was not significantly affected by dietary treatments. Regarding gastrointestinal tract characteristics, pH value of different parts of GI tract were significantly affected by dietary treatments. Villi height in duodenum by probiotic and in ileum by mannan oligosaccharide significantly increased. Villi width in duodenum by antibiotic and probiotic and in ileum by mannan oligosaccharide significantly increased. The number of goblet cells in duodenum by addition of antibiotic and in ileum by mannan oligosaccharide significantly increased. It was concluded that the use of organic acids and mannan oligosaccharide could have positive effects on performance of laying hens.

## Introduction

The poultry sector is continuously searching for new feed additives, in order to improve the feed efficiency and the animal health. The use of feed additives has two objectives: to control the pathogen microorganisms such as *Salmonella *and coliforms, and also to enhance the digestive microflora with beneficial microorganism.^1^ Although antibiotics possess these beneficial effects, their use as growth promoters in the poultry industry has been intensively controversial because of the development of bacterial resistance and potential consequences on the human health. Therefore, different compounds have been studied as natural and safe alternatives to antibiotics. In this regard, probiotics, prebiotics and organic acids have been suggested as the most important replacement candidates.^2^ Organic acids and their salts are generally regarded as safe (GRAS) and have been approved by most member states of EU to be used as the feed additives in animal production. Dietary organic acids and their salts are able to inhibit microorganisms growth in the food, and consequently to preserve the microbial balance in the gastrointestinal (GI) tract. In addition, by modifying intestinal pH, organic acids also improve the solubility of the feed ingredients, digestion and absorption of nutrients.^3^^,^^4^ The US national food ingredient association defined probiotic (direct fed microbial) as a source of live naturally occurring microorganisms, and this includes bacteria, fungi, and yeast.^5^ Probiotics are live microorganisms which will have beneficial effects on the host animal by improving its intestinal microbial balance through inhibiting intestinal pathogens. The mode of actions of probiotic is still unclear. However, some suggestions include: 1) beneficial changes in gut flora with reductions in the population of *Escherichia coli*, 2) lactate production with subsequent changes in intestinal pH, 3) production of antibiotic type substances, 4) production of enzymes, 5) competition for adhesion receptors in the intestine, 6) competition for nutrients, 7) reduction of toxin release and immune stimulation.^6^ Prebiotics are non-digestible feed ingredients that have selective effects on the intestinal microflora. They are consisting of nondigestible oligosaccharides which include fructo oligosaccharide, galacto oligosaccharide, trans galacto oligosaccharide and mannan oligosaccharide (MOS).^7^ Derived from the cell wall of *Saccharomyces cerevisiae *is neither hydrolysed by endogenous digestive enzymes nor absorbed by host, and it is considered as an prebiotic agent. It has been claimed that the benefits of MOS based on its specific properties such as modification of the intestinal flora, reduction in turnover rate of the intestinal mucosa and modulation of the immune system. These properties have the potential to enhance growth rate, feed efficiency and live ability in commercial broiler and turkeys, and egg production in layers.^8^

Eggshell quality is one of the most important issues in the poultry industry, influencing the economic profitability of egg production and hatchability. Besdies, high breaking strength of eggshell and absence of shell defects are essential for protection against the penetration of pathogenic bacteria such as *Salmonella *sp. into the eggs. It has been estimated that eggs with damaged shells account for 6.0 to 10.0% of all eggs produced, which leads to great economic loss.^9^^,^^10^ One of the main concerns is a decrease in eggshell quality is due to an increase in egg weight without an increase in the amount of calcium carbonate deposited in the shells. For this reason, the incidence of cracked eggs could even exceed 20.0% at the end of the laying period.^11^

The intestinal epithelial layer constitutes a barrier that protects the host against luminal pathogens.^12^ Reduced epithelial cell proliferation and mucosal atrophy of the intestine allow various pathogens invade the intestinal lumen. Feed additives such as antibiotics, probiotics, prebiotics and organic acids can help intestinal tissue fight against pathogens decreasing their population.^13^

The main objective of the study was to determine the performance, egg shell quality, pH values of some GI tract segments and small intestinal morphology of laying hens fed with antibiotic, organic acid, probiotic and prebiotic.

## Materials and Methods

**Birds and treatments.** One hundred and sixty 32 week old, Leghorn Hy-line W36 white layer hens were used in the study. Laying hens were weighed and randomly divided into five treatments. Each treatment consisted of four replicates of eight hens with equal mean body weight (experiment duration was 10 weeks). The experimental diets were formulated to contain all required nutrients of laying hens according to Hy-line w36 strain management manual. The ingredients and chemical composition of the basal diet are shown in Table 1. All diets were similar in energy, protein and other nutrients contents (Crude protein = 16.3%, ME = 2840 kcal kg^-1^). The experimental diets were T_1_: basal diet, T_2_: basal diet + 150 g per ton antibiotic (Oxytetracycline; Damloran Co., Tehran, Iran), T_3_: basal diet + 3 kg per ton of organic acid supplementation (Orgacid; Sunzen Biotech, Shah Alam, Malaysia), T_4_: basal diet + 50 g per ton probiotic (Protoxin; Probiotic International Limited, Somerset, UK) and T_5_: basal diet + 2 kg per ton of prebiotic (Mannan oligosaccharide; Biochem GmbH, Karlsruhe, Germany). Trade name of organic acid mixture was orgacid and contained mixture of formic, lactic, malic, citric, tartaric, and ortho phosphoric acids. This supplement contains 38.0% organic acids and 62.0% silicate as carriers. Probiotics used in the experiment was protoxin which included seven species of beneficial bacteria of the GI tract and two species of fungi.

**Table 1:** Ingredients and nutrient composition of the basal diet

**Ingredients**	**Percentage**
**Corn (%)**	58.75
**Soybean meal (%)**	25.70
**Soybean oil (%)**	3.32
**Oyster shell (%)**	5.07
**Limestone (%)**	4.00
**Dicalcium phosphate (%)**	2.13
**Vitamin-mineral premix (%)**	0.50
**Salt (%)**	0.30
**DL-Methionine (%)**	0.21
**Lysine (%)**	0.02
***Nutrient composition***	
**Metabolizable energy (kcal kg** ^-1^ **)**	2840
**Crude protein (%)**	16.30
**Calcium (%)**	4.00
**Total phosphorus (%)**	0.50
**Methionine (%)**	0.27
**Lysine (%)**	0.86
**Methionine + Cysteine (%)**	0.75
**Threonine (%)**	0.60
**Tryptophan (%)**	0.22

Provided each kilogram of vitamin and mineral premix: Vitamin A 7.040 g, Vitamin B_1 _0.591 g, Vitamin B_2 _1.600 g, Vitamin B_3 _3.136 g, Vitamin B_5 _13.860 g, Vitamin B_6 _0.985 g, Vitamin B_9 _0.192 g, Vitamin B_12 _0.004 g, Vitamin D_3 _2.000 g, Vitamin E 8.800 g, Vitamin K_3 _0.880 g, Vitamin H_2 _0.060 g, Choline chloride 80.000 g, Antioxidant 0.400 g, Mn 29.760 g, Fe 30.000 g, Zn 25.870 g, Cu 2.400 g, I 0.347 g, Se 0.080 g

Bacterial strains, included: *Lactobacillus acidophilus*, *L. Rhamnosus*, *L. Plantarum*, *Bifidobacterium*, *Enterococcus faecium*, *Streptococcus thermophilus *and yeast strains, including *Aspergillus oryzae *and *Candida pintolopesii*. One gram of this product contains at least 2 ×10^9^ bacteria.

Mannan oligosaccharide used as a prebiotic was separated from the outside wall of the yeast of *saccharomyces cerevisiae*. Additives were homogenously mixed with diet ingredients.

**Performance.** Body weights of laying hens were determined at the beginning (32 weeks of age) and end of the study (42 weeks of age). Egg production and egg weight were recorded daily throughout the study. Feed conversion was calculated as the ratio of gram of feed consumed per g of egg weight product. 

**Egg shell quality.** At the end of the experiment, three eggs from every replicate were selected and egg shell quality parameters such as shell percent, shell thickness and shell strength were measured. Shell thickness was measured at three locations on the eggs by micrometer (air cell, equator and sharp end) and mean value of measurements were reported. Egg shell strength was measured using egg shell tester equipment (Model OSK 13473; Fujiwara, Ogawa Seiki Co. Ltd., Tokyo, Japan) and was measured as a unit of compression force was exposed to a unit of eggshell surface area. 

**pH value and histological parameter.** At the end of the experimental period two hens from each replicate (eight hens from each treatment) were randomly selected and slaughtered by cervical dislocation. Internal organs of the GI tract were removed and all segments were identified. The value of pH for different segments of the GI tract was measured immediately by using a digital pH meter. To determine the pH, 10 g of contents from crop, gizzard, duodenum, jejunum, ileum and rectum were collected aseptically in 90 mL sterilized physiological saline (1: 10 dilution) and their pH were determined.^14^ Intestinal histology measurements were done according to the method of Yu *et al*.^15^ Sample sections (3 cm in length) were taken from the descending duodenum, the middle region of the jejunum, and the ileum region. Intestinal tissue samples were fixed in formalin and dehydrated, cleared, impregnation with paraffin. The processed tissue was then embedded in paraffin wax. Section were cut (6 μm) from the waxed tissue on LEICA RM 2145 microtome, cleared of wrinkles by floating on warm water (55 to 60 ˚C) prior to mounting on 10.0% poly-L-lysine coated slides. The slides were stained by haematoxylin and eosin. Histological indices were determined by use of a computer aided light microscopic image analyzer (Motic Images, 2000 1.2, Scion Image, Tokyo, Japan). The villous height, crypt depth were measured and calculation was made for villous height/crypt depth rate. 

**Statistical analysis.** The data were subjected to analysis of variance (ANOVA) using the General Linear Models (GLM) procedures of SAS software (Version 9.1; SAS Institute, Carry, USA) and the corresponding means were compared by Tukey-Kramer test at *p* < 0.05. The statistical model was as follows:

*Y*_ij_ *= µ + T*_i_ *+ E*_ij_

where Y_ij_ is the individual observation, µ is the experimental mean, T_i_ is the effect of experimental diet and E_ij _is the error term.

## Results

**Performance**. The performance parameters including body weight, egg weight, egg production and feed conversion ratio were shown in Table 2. There were no significant differences (*p* > 0.05) among experimental groups in body weights. Average egg weight significantly affected by added supplements so that groups received organic acid and mannan oligosaccharide significantly had higher egg weight compared to the control group (*p* < 0.05). Although egg production increased in group receiving mannan oligosaccharide, this increase was not significant. Hens received mannan oligosaccharide significantly improved feed conversion ratio compared to hens fed the control diet (*p* < 0.05).

**Table 2:** The effect of experimental treatments on production performance of laying hens

**Treatments**	**Initial body weight ** **(kg)**	**Final body weight (kg)**	**Feed conversion ratio (gr/gr)**	**Egg production (%)**	**Egg weight ** **(g)**
**Control** 1	1.450	1.517	2.020^a^	81.740	57.830^b^
**Control + Antibiotic** 2	1.460	1.535	1.990^a^	83.630	58.880^ab^
**Control + Organic acid** 3	1.450	1.557	1.960^a^	81.800	60.270^a^
**Control + Probiotic** 4	1.442	1.492	1.950^ab^	83.130	58.450^ab^
**Control + Prebiotic** 5	1.402	1.500	1.830^b^	86.870	60.020^a^
**SEM**	0.027	0.031	0.028	1.867	0.443
***p*** **-value**	0.634	0.588	0.003	0.330	0.019

a,b Different superscript indicate significant differences within each column (*p* < 0.05).

1 Control,

2 oxytetracycline (150 ppm),

3 orgacid (3 kg per ton of feed),

4 protoxin (50 ppm

5 mannan oligosaccharide (2 kg per ton of feed).

**Egg shell quality**. Analysis of the egg shell percentage, egg shell thickness and egg shell strength data are shown in Table 3. Egg shell percentage changes were not significantly different among treatments but usage of organic acid numerically increased it. Also added supplements had no significant effects on eggshell thickness and eggshell strength but egg shell strength showed a tendency to improve by mannan oligosaccharide.

**pH value of GI tract**. The effects of added supplements on acidity of the GI tract segments are shown in Table 4. The results indicated that organic acid caused significant decrease acidity of crop (*p* < 0.05). There was no significant difference in acidity of the proventriculus and gizzard among the experimental groups. Organic acid significantly decreased the acidity of duodenum and rectum compared to the control group (*p* < 0.05).

**Fig. 1:**
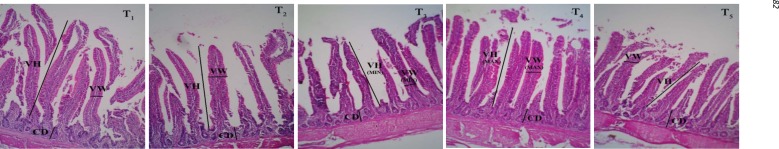
Histological figure of layer duodenum for different treatment groups, (H & E, 100×). T1: control. T2: antibiotic. T3: organic acid. T4: probiotic. T5: prebiotic. VH: villus height. VW: villus width. CD: crypt depth. MIN: minimum. MAX: maximum

**Fig. 2:**
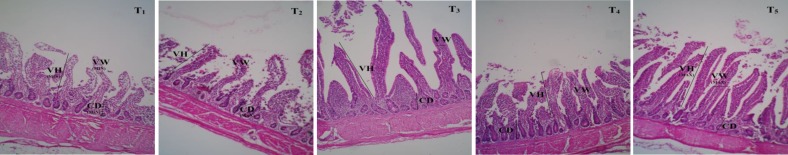
Histological figure of layer ileum for different treatment groups, (H & E, 100×). T1: control. T2: antibiotic. T3: organic acid. T4: probiotic. T5: prebiotic VH: villus height. VW: villus width. CD: crypt depth. MIN: minimum. MAX: maximum.

**Fig. 3:**
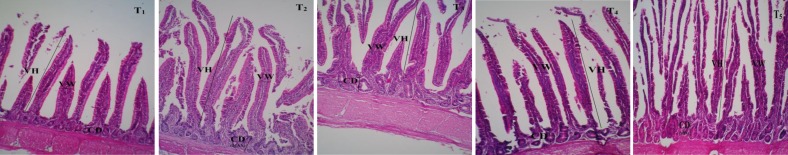
Histological figure of layer jejunum for different treatment groups, (H & E, 100×). T1: control. T2: antibiotic. T3: organic acid. T4: probiotic. T5: prebiotic VH: villus height. VW: villus width. CD: crypt depth. MIN: minimum. MAX: maximum.

**Histological findings. **The effects of added supplements on intestinal histomorphology change are shown in Table 5. The results showed that birds fed the diet containing probiotic had higher villi height in the duodenum (Fig. 1) than birds fed other additives (*p* < 0.05). Villi height in ileum (Fig. 2) by addition of mannan oligosaccharide to the diet significantly increased compared to control group (*p* < 0.05). In duodenum, addition of antibiotic and probiotic to the diet significantly increased villi width in comparison with group having organic acid (*p* < 0.05). Jejunum villi width was not significantly affected by added supplements but addition of mannan oligosaccharide significantly increased ileum villi width compared to the control group (*p* < 0.05). Also, no significant differences were observed between experimental groups regarding crypts depth in duodenum, while addition of mannan oligosaccharide to the diet significantly decreased crypts depth in jejunum (Fig. 3) and ileum, by compared to group receiving antibiotic (*p* < 0.05). Addition of antibiotic significantly increased the number of goblet cells in the duodenum compared to the organic acid group (*p* < 0.05). Furthermore, no significant differences were observed between experimental groups in the number of goblet cells of jejunum, but addition of mannan oligosaccharide significantly increased their population in ileum compared to control group (*p* < 0.05).

**Table 3:** The effect of experimental treatments on egg shell quality of laying hens

**Treatments**	**Egg shell percentage (%)**	**Egg shell thickness (mm)**	**Egg shell strength (kg per cm** ^2^ **)**
**Control** 1	11.440	0.365	0.380
**Control + Antibiotic** 2	11.580	0.360	0.341
**Control + Organic acid** 3	11.860	0.368	0.394
**Control + Probiotic** 4	11.590	0.356	0.370
**Control + Prebiotic** 5	11.680	0.373	0.395
**SEM**	0.222	0.006	0.018
***p*** **-value**	0.768	0.399	0.219

1 Control,

2 oxytetracycline (150 ppm),

3 orgacid (3 kg per ton of feed),

4 protoxin (50 ppm) and

5 mannan oligosaccharide (2 kg per ton of feed).

**Table 4:** The effect of experimental treatments on pH values of gastrointestinal tract segments in laying hens

**Treatments**	**Crop**	**Proventriculus**	**Gizzard**	**Duodenum**	**Jejunum**	**Ileum**	**Rectum**
**Control** 1	5.800^ab^	5.630	4.760	5.670^ac^	5.810^ab^	6.640	6.330^ab^
**Control + Antibiotic** 2	5.860^a^	5.340	4.710	5.450^b^	5.810^ac^	6.020	6.220^b^
**Control + Organic acid** 3	5.270^b^	5.300	4.810	5.420^b^	5.560^b^	5.760	5.840^c^
**Control + Probiotic** 4	5.900^a^	5.670	7.890	5.550^bc^	5.610^bc^	5.910	6.150^bc^
**Control + Prebiotic** 5	5.690a^b^	5.410	4.850	5.750^a^	5.910^a^	5.940	6.630^a^
**SEM**	0.135	0.101	0.117	0.034	0.057	0.344	0.076
***p*** **-value**	0.030	0.070	0.845	0.0001	0.002	0.443	0.0001

a,b Different superscript indicate significant differences within each column (*p* < 0.05).

1 Control,

2 oxytetracycline (150 ppm),

3 orgacid (3 kg per ton of feed),

4 protoxin (50 ppm) and

5 mannan oligosaccharide (2 kg per ton of feed).

**Table 5:** The effect of treatments on small intestines histomorphology of laying hens (μm).

**GI Segment**	**Treatment**	**Villus height**	**Villus width**	**Crypt depth**	**Villus height** **/** ** Crypt depth**	**Goblet cells**
***Duodenum***						
	**Control** 1	825.000^bc^	94.500^ab^	325.000	2.560^ab^	13.500^ab^
	**Control + Antibiotic** 2	1127.250^ac^	102.250^a^	385.000	2.910^a^	15.000^a^
	**Control + Organic acid** 3	667.500^b^	77.250^b^	315.000	2.150^b^	9.500^b^
	**Control + Probiotic** 4	1170.000^a^	105.000^a^	375.000	3.100^a^	12.500^ab^
	**Control + Prebiotic** 5	800.000^b^	92.500^ab^	365.000	2.190^b^	11.500^ab^
	**SEM**	74.362	4.357	18.073	0.162	1.032
	***p*** **-value**	0.0008	0.0037	0.054	0.0025	0.0205
***Jejunum***						
	**Control** 1	690.000	98.370	350.000^ab^	1.980	12.000
	**Control + Antibiotic** 2	880.000	105.250	440.000^a^	2.020	11.500
	**Control + Organic acid** 3	575.000	97.500	300.000^ab^	2.000	11.000
	**Control + Probiotic** 4	915.000	85.250	385.000^ab^	2.390	12.000
	**Control + Prebiotic** 5	765.000	94.500	275.000^b^	2.730	12.500
	**SEM**	109.818	7.438	34.472	0.275	0.816
	***p*** **-value**	0.224	0.460	0.028	0.273	0.744
***Ileum***						
	**Control** 1	410.000^b^	86.000^b^	205.000^b^	1.990^ab^	9.500^b^
	**Control + Antibiotic** 2	560.000^ab^	87.500^ab^	275.000^a^	2.030^ab^	11.500^ab^
	**Control + Organic acid** 3	500.000^ab^	88.000^ab^	215.000^bc^	2.300^ab^	10.500^ab^
	**Control + Probiotic** 4	460.000^ab^	88.750^ab^	245.000^ac^	1.860^b^	10.000^b^
	**Control + Prebiotic** 5	600.000^a^	95.500^a^	230.000^bc^	2.610^a^	13.500^a^
	**SEM**	40.424	1.944	8.530	0.144	0.730
	***p*** **-value**	0.0318	0.0300	0.0003	0.0167	0.0118

a,b,c Different superscript indicate significant differences within each column (*p* < 0.05).

1 Control,

2 oxytetracycline (150 ppm),

3 orgacid (3 kg per ton of feed),

4 protoxin (50 ppm)

5 mannan oligosaccharide (2 kg per ton of feed).

## Discussion

Previous studies reported that organic acids such as fumaric, propionic, butyric and their salts have variable effects on egg production and egg quality traits. These discrepancies would be related to the source and amount of organic acids, environmental condition and the composition of the diets.^16^ The beneficial effect of ascorbic acid supplementation upon egg weight, during the hot season, has also been reported in white Leghorn by Perek and Kendler.^17^^,^^18^ However, many researchers reported that egg weight was not affected by use of organic acids additives.^16^^,^^19^^-^^22^ Significant effect of organic acid on average egg weight that observed in this experiment is in agreement with Langhout and Sus who observed heavier eggs by use of organic acids supplementation.^23^ Researchers found no effect of MOS on egg weight in laying quail.^24^ On the other hand, Gracia *et al*. reported that egg weight significantly increased from 54 to 58 weeks with a tendency of increment up to 62 week of age by adding MOS to the diet, but they did not find any positive effects thereafter.^25^ Gibson and Roberfroid indicated that prebiotic can beneficially affect the host by selectively stimulating the growth, and/or activity of healthy bacteria in the colon.^26^ Prebiotics, such as inulin or oligofructose, have been shown to change the intestinal microflora and suppress the undesirable bacteria^27^^,^^28^ and stimulate mineral absorption, mainly calcium and magnesium.^29^

However, it is of interest to note that there are few reports available related to the effects of prebiotics on egg-laying performance. In this study, MOS supplementation to the diet of laying hens significantly increased egg weight and feed conversion ratio compared to the control. On the basis of our results, improvement in egg weight and feed conversion ratio might be due to healthier birds whose feed efficiency and mineral absorption have been improved by organic acid and mannan oligosaccharide. Improved feed conversion may be the result of the recovery of damaged cells of the digestive wall and preservation of microbial balance and improved nutrient utilization of hens belongs to supplemented groups. It can be explained that prebiotics helped colonization of beneficial microbial flora in the GI tract and prevented colonization of pathogenic bacteria. Subsequently, hens received prebiotics had healthier gut and consequently better performance.

Egg shell quality parameters were not affected by the supplements. The results of some studies carried out on rats, broiler chickens and pigs have indicated that organic acids may improve the utilization of minerals in monogastric animals.^30^^-^^35^ One of the mechanism of this effect is connected with the reduction of intestinal pH, which leads to an increase in the activity of digestive enzymes (accelerated conversion of pepsinogen to pepsin), and in the solubility of minerals. Increase shell thickness was reported by Soltan who found improved egg shell thickness by use of organic acids supplementation.^21^ Contradictory result was found by Yesilbag and Colpan who reported organic acid mixture did not improve shell thickness.^22^ The authors indicated that the observed improvement in eggshell quality was connected with an increase in Ca concentration in serum, which could be attributed to the beneficial effect of organic acids on Ca absorption.^21^ The differences in egg shell quality maybe a consequence of the increased mineral and protein absorption.^21^ The phenomenon of increased absorption is reflected in the increased calcium and protein deposits of the shell and contributes to improving the quality which may result in increased shell weight and thickness.

Current study data indicated that pH value of GI tract segments significantly decreased by use of organic acid. Florou-Paneri *et al*. did not observed any change in pH of the GI tract when used a mixture of organic acids in broiler chickens.^36^ On the other hand Clik and Ersoy reported that pH of crop and duodenum decreased by addition of organic acid into the diet.^37^ Adding beneficial bacteria as probiotics and indigestible oligosaccharides reduced GI pH and disrupted the environment for salmonella and bacillus that their optimal pH was about 7,^38^ thus improving performance, feed conversion and growth rate in birds.^39^ Production of short-chain fatty acids (such as acetate, propionate and butyrate) and lactic acid resulting from the fermentation of inulin, reduces the acidity of the gut which provides favorable conditions for the growth of lactic acid bacteria.^40^ Decreasing pH in GI tract had a beneficial effect on the inhibition of intestinal bacteria competition with the host for available nutrients and the possibility of reducing bacterial toxicity, e.g., ammonia and amines, thus improving weight gain of the host animals. Furthermore, the growth inhibition of potential pathogen bacteria, e.g., *E. coli* and *Salmonella*, in the feed and GI tract is beneficial to animal state of health.^41^ Organic acid additives in this experiment decreased the pH of different parts of the GI tract and given that acidification of the digestive tract by effect on beneficial bacteria improved digestion and absorption of nutrients so can conclude that these factors are one of the reasons for improve the performance of laying hens by adding organic acid.

Intestinal morphology characteristics are affected by dietary treatments. In this study the results showed that the use of probiotic and prebiotic improved intestinal morphology characteristics, which this reaction could increase feed utilization and improve performance. Overall, gut surface area affects net utilization of dietary nutrients in birds and is determined by gross morphological features such as length and cross-sectional area of the duodenal, jejunal and ileal segments, and by finer morphological features such as villi height and surface area of the epithelium in each of those segments.^42^ Good intestinal health in the poultry industry is of great importance to achieve target growth rates and feed efficiency.^43^ Antimicrobial agents are known to reduce the intestinal pathogenic microbial load reducing the presence of toxins which are associated with changes in intestinal morphology, such as shorter villi and deeper crypts.^44^ The intestinal epithelial layer constitutes a barrier that protects the host against luminal pathogens.^12^ Reduction in epithelial cell proliferation and mucosal atrophy of the intestine allow various pathogens to invade in the intestinal lumen. Feed additives such as antibiotics, probiotics or organic acids can help intestinal tissue decrease the pathogens.^13^ According to Klasing intestinal mucus layer thickness generally from the beginning to the end of it gradually declined and also villi height and crypt depth decreases,^45^ that is in agreements with results of this study. Length and width of intestinal villi are of histomorphometrical indices and any increase in the values enhances the absorptive surface of intestine. Researchers have shown that organic acids can reduce the intestinal lumen pH and increase antibacterial enzymes produced by some bacteria, so increasing villi height.^46^ Moreover, organic acids reduce amount of pathogenic bacteria in the small intestine wall and decreases production of toxic compounds which cause changes in the morphology of the intestine of birds and in consequence prevent destruction and damage to intestinal epithelial cells.^25^ On the other hand ineffectiveness of organic acids on villi height observed in our experiment has also been reported by Vieira *et al*.^47^ They indicated that the addition of a blend of organic acids did not affect villus height or crypts depth on broilers. Some information on the gut health could be obtained by studying the structure of the intestinal mucosa.^44^ Villus condition is a common criteria measurement for investigation of the effects of nutrition on gut physiology. Longer villus could be considered as an indicator of an active functioning of intestinal villi. Increased villi height provides more surface area for nutrients absorption.^48^ However, in many cases significant correlations were not observed between performance and villus height or crypt depth.^47^ Therefore, the positive effects of organic acids on performance that was observed in this experiment may be due to reduced pH of the gastrointestinal tract and thereby reducing the harmful bacteria. It has been reported that probiotics increase short chain fatty acids (SCFA) and decrease the production of ammonium.^49^ These fatty acids can reduce the pH of small intestine and improve beneficial microbial population of gut. When probiotics are consumed, a large amount of useful microorganisms enters the animal’s gastrointestinal tract. These microorganisms produce acids (such as acetic acid and lactic acid) and other compounds that inhibition growth of pathogenic bacteria and aid beneficial bacteria to adhere and rapidly colonize the intestinal mucosa of the animal.^50^ As mentioned, width and height of the villi in the ileum increased by use of mannan oligosaccharide. Changes in villus height due to the supplementation with prebiotics have been reported previously. Baurhoo *et al*. found that birds fed diet containing prebiotic had longer villi than those fed the control diet.^51^ Pelicano *et al*. reported an increase in jejunal villi length in broiler fed combination of mannan oligosaccharide and organic acid.^52^ In a study conducted by Xu *et al*,^44^ dietary addition of a prebiotics significantly increased villus height. They suggested that these changes may be related to the ability of prebiotic to create a more favorable intestinal microbial environment and are not a direct effect of prebiotic on the intestinal tissue. In the current study, addition of prebiotic had beneficial effects on performance and intestinal morphology. Positive effects of prebiotics could be related to their inhibitory effects on intestinal pathogens. It has previously been reported that prebiotics are able to control pathogenic or potential pathogenic bacteria which possess type-1 fimbriae, resulting in better performance.^53^ It has also been reported that presence of toxins in gut can cause some changes in intestinal morphology (shorter villi and deeper crypts).^48^ This reduction in villus height can reduce nutrient absorption due to decreased intestinal surface area for absorption. Diarrhea, which is a consequence of deeper crypt and shorter villi, decreases resistance to disease and lowers growth performance and increases secretion of gastrointestinal.^44^ Therefore, we concluded that enhancements of villus height and reduction of crypts depth and high ratio of villus height/crypt depth were paralleled with increased digestive and absorptive capacity of the small intestine. Apart from pH and microbiological profile of the gut, histological changes of the small intestine might influence the performance of the birds in this study. 

In conclusion, according to results of this experiment it could be recommended that probiotics, prebiotics and organic acids could be used as antibiotic alternatives in layers feed.
